# Role of sex hormones in diabetic nephropathy

**DOI:** 10.3389/fendo.2023.1135530

**Published:** 2023-04-18

**Authors:** Jiahui Liu, Zhe Liu, Weixia Sun, Ling Luo, Xingna An, Dehai Yu, Wanning Wang

**Affiliations:** ^1^ Public Research Platform, First Hospital of Jilin University, Changchun, Jilin, China; ^2^ College of Basic Medical Sciences, Jilin University, Changchun, Jilin, China; ^3^ Nephrology Department, First Hospital of Jilin University, Changchun, Jilin, China

**Keywords:** sex hormones, estrogen, testosterone, diabetic nephropathy, metabolism

## Abstract

Diabetic nephropathy (DN) is the most common microvascular complication in diabetes and one of the leading causes of end-stage renal disease. The standard treatments for patients with classic DN focus on blood glucose and blood pressure control, but these treatments can only slow the progression of DN instead of stopping or reversing the disease. In recent years, new drugs targeting the pathological mechanisms of DN (e.g., blocking oxidative stress or inflammation) have emerged, and new therapeutic strategies targeting pathological mechanisms are gaining increasing attention. A growing number of epidemiological and clinical studies suggest that sex hormones play an important role in the onset and progression of DN. Testosterone is the main sex hormone in males and is thought to accelerate the occurrence and progression of DN. Estrogen is the main sex hormone in females and is thought to have renoprotective effects. However, the underlying molecular mechanism by which sex hormones regulate DN has not been fully elucidated and summarized. This review aims to summarize the correlation between sex hormones and DN and evaluate the value of hormonotherapy in DN.

## Introduction

Diabetic nephropathy (DN) is one of the most common and serious complications of diabetes mellitus and a major cause of chronic kidney disease and end-stage renal disease (ESRD) (1–4). The occurrence and progression of DN are closely related to patient blood glucose levels, blood pressure, genetic background and age (5, 6). Unlike other renal diseases, once macroalbuminuria occurs, DN will remain throughout life, which makes DN a major cause of death in patients with diabetes. DN patients at the end stage of renal failure rely on dialysis and kidney transplantation. Therefore, preventing and treating DN has become a pressing problem worldwide. Many studies have shown that the occurrence and development of DN are closely correlated with sex (7). In addition to social roles, psychological cognition and behavioral habits, the most important difference between the sexes is sex hormones. Especially in women, sex hormones vary greatly throughout life, from infancy to adolescence, sexual maturity, pregnancy, perimenopause and postmenopause. However, the underlying molecular mechanism by which sex hormones regulate DN has not been fully elucidated. Moreover, based on the impact of sex hormone imbalances on the development of DN, hormone therapy in patients with diabetes may alleviate diabetic kidney injury to a certain extent and is a potentially valuable therapeutic strategy for DN patients.

In this article, we summarized the effects of sex hormone changes on DN development by searching and reviewing published articles. We hope our work will provide information on the correlation between sex hormones and DN and provide new clues for the treatment of DN.

## Sex hormones

Sex hormones are steroidal hormones synthesized mainly by the gonads, the placenta, and the reticular cortex of the adrenal gland in animals. In female animals, the ovaries mainly secrete two types of sex hormones: estrogen and progesterone. In male animals, the testes secrete androgens, mainly testosterone.

**Figure 1:**
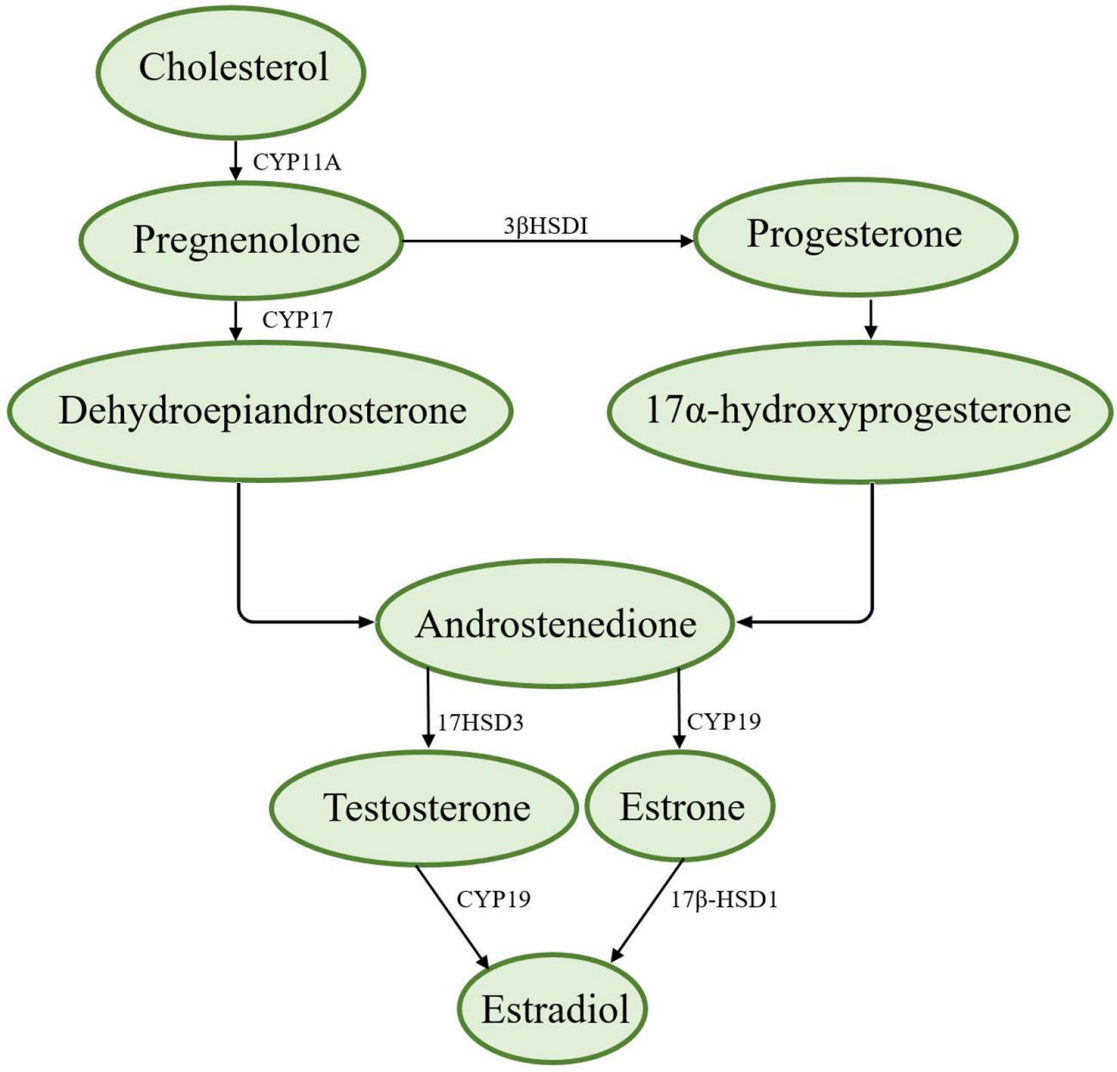
Synthesis of sex hormones.

The synthesis of sex hormones is based on cholesterol, which is converted to pregnenolone by cytochrome P-11A (CYP11A). Pregnenolone can be converted to progesterone by 3βHSDI and transported from the outer mitochondrial membrane to the inner mitochondrial membrane by transporters (8). There are two ways to synthesize androstenedione. First, pregnenolone is converted to dehydroepiandrosterone by CYP17 and then to androstenedione; second, progesterone is converted to 17α-hydroxyprogesterone and then to androstenedione (8). Androstenedione is converted to testosterone by the enzyme 17HSD3, which is converted to estradiol *via* aromatase (CYP19) (8, 9). **Figure 1** shows the synthesis of sex hormones.

Most sex hormones are metabolically inactivated in a similar manner: by forming more water-soluble conjugates, such as glucuronides or sulfate esters, in metabolic organs, such as the liver and kidneys. These conjugates are then excreted in urine or secreted into the intestine with bile and excreted in feces (10, 11).

## Testosterone

Testosterone is a steroid hormone. It is the main sex hormone and anabolic hormone in the male body and is mainly synthesized by the interstitial cells of the testicles. Other organs, such as the adrenal glands and ovaries, also produce small amounts of testosterone.

Androgen receptor (AR) is encoded by the AR gene on the X chromosome and is widely distributed in various tissues and organs, including the endothelium and kidney (12). AR plays an important role in the development and maintenance of the reproductive, musculoskeletal, cardiovascular, immune, neurological and hematopoietic systems (12, 13). When not bound to testosterone, AR is bound in the cytoplasm by heat shock protein (HSP) and chaperone proteins. When interacting with testosterone or dihydrotestosterone, AR is released from HSP and chaperone proteins and translocates to the nucleus to produce the corresponding biological effects (14). Sankar et al. reported that AR was a key determinant of the response to testosterone, and circulating levels of testosterone can influence spatial cognition in adult males (15).

## Estrogen

Estrogens are produced by the placenta and ovaries of female animals and promote the development of secondary sexual characteristics and the maturation of sexual organs in females. There are three main types of estrogens in females, estrone (E1), estradiol (E2) and estriol (E3). These estrogens play important roles in regulating many physiological functions, such as cell proliferation and differentiation, development, body homeostasis and metabolism (16–20). Under physiological and pathophysiological conditions, the effects of estrogen are mediated by estrogen receptors α/β and G protein-coupled estrogen receptors (GPER). These receptors are involved in the development of many diseases, including DN, cancer, neurodegenerative diseases, and cardiovascular, metabolic and autoimmune diseases (21–24).

## Alterations in sex hormones in diabetes

### Alterations in sex hormones between the sexes

Under physiological conditions, sex hormone levels and their functions in men and women alter with increasing age. Both testosterone and estrogen have been found to decline with age in men and women (25–28). Gambineri et al. summarized the reasons for the difference in circulating sex hormone levels between the sexes, and they believed that it is due to the difference in the synthesis site of sex hormones, the conversion rate of sex hormones to each other, and the binding degree of sex hormones to sex hormone binding globulin (SHBG) in the two sexes (29).

### Alterations in sex hormones in diabetes

Diabetes can cause an imbalance in sex hormones in patients (30). Studies have shown that compared with men without diabetes, men with diabetes have decreased levels of testosterone and increased levels of E2. However, testosterone levels are higher and E2 levels are lower in women with diabetes than in those without diabetes, suggesting that diabetes is associated with an imbalance in sex hormones (30–37). In females with diabetes (compared with females without diabetes), the decreased level of E2 may reduce creatinine clearance and increase urine albumin excretion and tubular fibrosis in the kidney, which may increase the risk of developing renal complications (38).

Insulin levels have a significant impact on the functional regulation of the hypothalamic-pituitary-gonadal axis (HPGA) (39). Normally, insulin is secreted by pancreatic β cells. Then, it binds to insulin receptors and activates intracellular protein tyrosine kinase (PTK). Activated PTK can phosphorylate and activate insulin receptor substrates (IRS) to activate phosphoinositide 3-kinase (PI3K). The activated PI3K signaling cascade enhances gonadotropin-releasing hormone (GnRH) secretion in the hypothalamus, which stimulates the pituitary secretion of luteotropic hormone (LH) and follicle-stimulating hormone (FSH) and eventually induces the release of sex hormones by the gonads (40). Approximately 5% of sex steroids are present in the blood and enter cells through specific receptors on the plasmalemma (41). During diabetes, altered levels of SHBG, increased levels of oxidative stress and increased levels of CYP19 activity are present in adipose tissue. This results in the conversion of testosterone and androstenedione to estradiol and estrone, respectively, which contribute to reducing serum testosterone concentrations in men with diabetes (42–47). In addition, the disruption of glucolipid metabolism, the reduced bioavailability of insulin during diabetes and the reduced activity of CYP19 in the ovaries of diabetic rats, as determined by Bozkurt et al., might be responsible for the reduced levels of estradiol in females with diabetes (48–50).

The level of insulin can be affected by leptin. Leptin is a type of adipokine that is secreted by adipose tissue. It can regulate energy metabolism and may regulate reproductive function by regulating the release of GnRH in the hypothalamus (39, 51, 52). The level of insulin can also affect the generation of leptin (53, 54). Under normal circumstances, leptin phosphorylates IRS-2 on hypothalamic leptin receptors, activating PI3K and stimulating the release of GnRH (55, 56). Under diabetic circumstances, the feedback between insulin and leptin is disordered, thus impairing the release of GnRH and ultimately reducing sex hormone secretion.

In contrast, altered levels of sex hormones may be a predisposing factor for diabetes. CYP19 is the limiting enzyme for estradiol synthesis. Jones et al. found that in aromatase-knockout (ArKO) female mice, glucose oxidation was decreased and obesity and insulin levels were increased (57). A study showed that decreased CYP19 activity combined with low concentrations of dihydrotestosterone (DHT) downregulates the expression of transforming growth factor-β (TGF-β) and type IV collagen and inhibits the level of glomerulosclerosis and tubular interstitial fibrosis, thus attenuating the progression of renal complications in male diabetic rats (35). Takeda et al. showed that a short-term E2 treatment could reverse the development of glucose intolerance and insulin resistance by enhancing lipid metabolism in male ArKO mice (58).

## Role of sex hormones in the development of DN

### Sex differences in the development of DN

The occurrence and development of DN are affected by sex to a great extent (59–61). Observations in humans and animals showed that the level of sex steroids in males and females are altered by DN. Plasma testosterone levels in men were decreased to levels similar to those in women, while plasma estradiol levels in women were decreased to levels similar to those in men. In many DN models, male animals tend to progress more quickly than female animals. In type 1 and type 2 diabetes, the prevalence of microproteinuria and macroproteinuria is higher in males than in females, and the risk of microproteinuria and progression to macroproteinuria is also higher (7, 62–65). This phenomenon is also seen in nondiabetic renal diseases. Neugarten et al. found that men with chronic renal disease show a more rapid decline in renal function than women with chronic renal disease (66).

However, other studies showed an opposite result, as they reported that women with diabetes have a higher risk of progressing to ESRD than men with diabetes (67). When the women with diabetes included in the statistics were older (postmenopausal), they had a higher rate of progression to ESRD (68). In the Irbesartan DN trial and the angiotensin II (AngII) receptor antagonist Losartan study, postmenopausal women with diabetes developed end-stage renal disease at a faster rate than men with diabetes (69, 70). In addition, age at diagnosis of type 1 diabetes also has an impact on the timing of the onset of ESRD in both sexes. Men diagnosed with type 1 diabetes before puberty had a delayed onset of ESRD, while women diagnosed at puberty face a higher risk of ESRD (71, 72).

### Role of testosterone in DN

The risk of renal complications in men with diabetes is higher than that in premenopausal women with diabetes. Testosterone is considered to be more conducive to the genesis of DN in males. Kang et al. reported that men have a higher risk of renal complications (73). Sharon et al. reported that the decrease in testosterone may partly attenuate kidney injury in males (74). Jan et al. reported that men with type 1 diabetes have a higher risk of ESRD and mortality (75). In contrast, the effect of testosterone on the progression of DN in females with diabetes is rarely mentioned, and females are considered to be less influenced by testosterone (73).

### Role of estrogen in DN

Changes in estrogen levels affect the occurrence of DN, and estrogen may have different effects in males and females with diabetes (76, 77). As mentioned above, the level of circulating testosterone in men with diabetes is decreased, while the level of E2 is increased (33, 36, 37). The increased level of E2 may increase the risk of renal complications in men (38, 60, 78). In male STZ-induced diabetic rats, inhibition of testosterone transformation to estradiol attenuates inflammation and the expression of type IV collagen and TGF-β; hence, the progression of DN is reduced (78).

As the most important sex hormone in women, estrogen has been shown to prevent podocyte apoptosis. Estrogen can also inhibit type I/IV collagen synthesis in mesangial cells and promote the degradation of the extracellular matrix, which are critical factors that induce tubular fibrosis (79–81). The effect of estrogen on the female kidney may vary at the postmenopause stage. William et al. reported that women at the postmenopause stage have a higher risk of renal complications (70). Lewis et al. found that kidney function was reduced in women with diabetes with an average age of 58 (69). Studies have shown that women who undergo ovariectomy (OVX) have a higher risk of diabetes and other complications (82–84). Mankhey et al. reported that in STZ-induced diabetic female rats, OVX could enhance DN, whereas 17-β-estradiol replacement therapy could attenuate DN (38). Therefore, estrogen is considered to have a renal protective function in women with diabetes.

### Sex hormones affect the genesis of DN and its underlying mechanisms

Patients with diabetes who progress to nephropathy have significantly higher initial mean blood pressure, cholesterol, HbA1c, low-density lipoprotein (LDL) cholesterol and triglyceride levels (85). The development of DN includes renal hemodynamic changes, sugar/lipid metabolic disorders, and the effects of oxidative stress and inflammation. These changes cause glomerular basement membrane thickening, mesangial matrix accumulation, glomerular sclerosis and tubular epithelial cell injury, which eventually lead to renal tubular fibrosis, proteinuria and the leakage of large molecules (86–88).

#### ●Oxidative stress and inflammation

In the diabetic state, NADPH oxidases (Nox proteins) are activated to produce excess reactive oxygen species (ROS) through the electron transport chain (89). When too many ROS accumulate, they attack organs, including the kidney, and this is accompanied by the depletion of antioxidants. Additionally, the oxidative/antioxidant system balance is disrupted, resulting in oxidative stress (89, 90). The kidney contains a high density of mitochondria. Excess ROS lead to oxidative damage to mitochondrial proteins and mitochondrial DNA (mtDNA). Then, the kidney fails to filter and reabsorb Na+, glucose and other metabolites from the urine, and vascular permeability is increased (91, 92). Testosterone may reduce the activation of STAT3 to increase the production of ROS (93). Mustafa and Mehmet found that estradiol had positive effects on the antioxidant defense system and tissue lipid peroxidation in OVX diabetic rats, possibly by enhancing the antioxidant activities in the kidney, thus protecting against diabetes (94). Hong et al. found that estrogen can inactivate Nox, inhibit the production of superoxide anions, and reduce oxidative stress in the kidney, thus reducing kidney injury (95, 96).

The high glucose environment of diabetes also leads to increased production of advanced glycation end products (AGEs), which interact with their receptor RAGE to activate NF-κB. Then, inflammatory responses occur, producing multiple proinflammatory and profibrotic molecules (97–100). T and B lymphocytes are subsequently activated (101). Activated T lymphocytes can produce proinflammatory cytokines (e.g., IL-17, IL-6, TNF-α and IFN-γ) or recruit and activate macrophages (102–108). Activated B lymphocytes can induce the formation of inflammatory immune complexes and produce proinflammatory cytokines (e.g., IL-6, IL-10 and TNF) (106, 109–111). After proinflammatory cytokines are released, the cascade amplifies the NF-κB signal, produces more proinflammatory cytokines and recruits adjacent macrophages to the inflammatory site in tubules, which leads to kidney infiltration, increases the expression of proinflammatory and profibrotic molecules (e.g. type I/IV collagen and TGF-β), and exacerbates renal tubular fibrosis (101, 111).

In the diabetic state, testosterone can phosphorylate and activate C-jun (a molecule that functions in renal inflammation) (112–114). Activated C-jun may upregulate monocyte chemoattractant protein-1 (MCP-1) expression. This promotes tubular epithelial cells to attract macrophages to the injury site of tubules, causing local inflammation and tubular cell apoptosis. The activation of C-jun can also upregulate the expression kidney injury molecule-1 and directly induce tubular fibrosis (114, 115). In SD male rats, once inflammation occurs in the kidney, testosterone can upregulate the expression of the proinflammatory cytokine TNF-α to exacerbate the inflammatory response and increase the expression of profibrotic substances to promote tubule epithelial-mesenchymal transition (EMT) and promote renal fibrosis (116).

Tubular fibrosis is the outcome of the inflammatory response in the kidney and is led by TGF-β (a key molecule that can stimulate the production of several extracellular matrix proteins that accumulate in the diabetic kidney, including type IV collagen, fibronectin and laminin). EMT of the renal tubular epithelium leads to tubular fibrosis (117, 118). In the state of diabetes, DHT upregulates the expression of TGF-β in diabetic male rats and accelerates the production of the early fibrosis marker connective tissue growth factor (CTGF). Additionally, epithelial cells acquire a fibroblast phenotype, leading to the genesis of tubular fibrosis (60).

Estrogen can interfere with the expression of TGF-β and its downstream signaling pathway *via* members of the small mother against decapentaplegic (Smad) protein family (Smad2/Smad3/Smad6/Smad7) (80, 119). Studies have shown that in STZ-induced diabetic female rats, E2 regulates the activity of TGF-β by downregulating profibrotic signaling molecules (Smad2, Smad3) and upregulating antifibrotic signaling molecules (Smad6, Smad7) (80). Thus, E2 can reduce proteinuria and ECM protein expression associated with diabetic glomerulosclerosis and renal tubular fibrosis and play a renoprotective role in females with diabetes (80). Regulation of casein kinase II (CK2) is another mechanism by which E2 may regulate TGF-β activity. CK2 is a serine/threonine protein kinase that, when activated, phosphorylates early growth reactivity 1 (EGR-1). EGR-1 typically binds to specific protein 1 (Sp1), preventing Sp1 from binding to target sequences. Ck2 induces EGR-1 phosphorylation in response to TGF-β to prevent the formation of the EGR-1/Sp1 complex, and the level of free Sp1 increases. Sp1, in turn, binds to target sequences in the promoters of type IV collagen and increases its synthesis. In murine mesangial cells, E2 treatment prevented the TGF-β-induced increase in CK2 expression and activity, thereby inhibiting TGF-β signaling and type IV collagen upregulation (120).

In addition to regulating TGF-β expression and activity in renal cells, E2 can also indirectly regulate TGF-β in the kidney by regulating macrophage infiltration. Macrophages are a key source of TGF-β in diabetic kidneys. In a spontaneously hypertensive rat model of kidney disease, the level of macrophage infiltration in the kidney was higher in males than in females, and OVX in females increased the number of macrophages. Similarly, OVX in diabetic female rats increased macrophage infiltration, and this effect could be normalized by E2 treatment (80, 121). These data suggest that E2 inhibits macrophage infiltration, thereby preventing the production of TGF-β by a major source and potentially protecting the kidney from injury.

#### ●Hemodynamic changes

Increases in ROS are generated by persistent hyperglycemia and can lead to dilatation of the afferent glomerular arteriole, hyperfiltration, hypertransfusion and high internal pressure in the kidney in the early stages of diabetes (122). A prolonged high filtration load due to high glucose increases sodium-glucose cotransporter protein 2 levels in the proximal tubules, and the resorption of glucose and sodium chloride increases. This leads to dysfunctional tubuloglomerular feedback and results in the disruption of the afferent/efferent arteriole balance and increased glomerular unit plasma flow (123). This abnormal status ultimately increases the renal glomerular filtration rate (GFR) and causes glomerulosclerosis.

Before adolescence, sex does not play a significant role in the incidence of DN (124). With aging and the occurrence of chronic complications associated with diabetes mellitus, DN tends to begin earlier in men than in women because testosterone can activate the renin-angiotensin-aldosterone system (RAAS) (73). The RAAS is one of the primary control systems that regulates the balance of blood pressure and fluids, and the kidney is the organ that activates the RAAS. The major bioactive hormone in the RAAS is AngII, which is cleaved from angiotensinogen and can promote vasoconstriction, fibrosis, inflammation and apoptosis (125–128). AngII receptors can be divided into two types according to their length: ATR1 (40 kDa) and ATR2 (41 kDa). ATR1 is considered to be associated with increased blood pressure and vasoconstriction, while AT2R is considered to be associated with reduced blood pressure and inflammation inhibition (127, 128). DHT upregulates ATR1 expression in sexually mature SD male rats (73). The activity of AngII might be modulated by angiotensin-converting enzyme 2 (ACE2) or 3β-HSD4 in males. ACE2 is a zinc metalloproteinase that may degrade AngII to Ang-(1-7) (128–130). Oudit et al. found that the loss of ACE2 exacerbated the degree of glomerulosclerosis in male mice (131). 3β-HSD4 is a ketone reductase whose activity is regulated by angiotensin; it can reduce testosterone and progesterone to inactive metabolites. Under normal conditions, 3β-HSD4 protects the kidney from the potential negative effects of testosterone; in patients with diabetes with increased AngII levels, the loss of 3β-HSD4 activity may increase the susceptibility of the kidney to testosterone-induced damage (132).

Estrogen has a regulatory effect on the RAAS. It can attenuate AngII-induced hypertension and reduce renal insufficiency (73, 130, 133–135). Nitric oxide (NO) can dilate blood vessels, and endothelial cells produce NO through endothelial nitric oxide synthase (eNOS) to regulate vascular tone (136). NO can counteract the vasoconstrictive effects of AngII (137). Acute hyperglycemia induces a state of oxidative stress in the endothelium, which reduces NO production and leads to endothelial dysfunction (137). Estrogen can upregulate eNOS expression to accelerate NO release or increase NO bioavailability to relax blood vessels and lower blood pressure, thereby reducing glomerular sclerosis (138–141). Estrogen can also stimulate NO release and attenuate glomerular sclerosis and renal fibrosis by upregulating ATR2 expression in the renal medulla (142).

#### ●Metabolic disorders

There are two aspects of abnormal glucose metabolism in patients with diabetes. AGEs bind to their receptors to activate the NF-κB pathway and stimulate the production of vascular endothelial growth factor (VEGF), TGF-β and MCP, leading to glomerular podocyte loss, expansion of the glomerular extracellular matrix and progressive glomerulosclerosis (143). Second, protein kinase C is activated by high glucose levels. This results in decreased production of eNOS and increased production of VEGF, which destabilize the endothelial microenvironment and activate the NF-κB pathway. The NF-κB-mediated inflammatory response leads to tubular fibrosis (99).

Persistent hyperglycemia in patients with diabetes can promote fatty acid synthesis and triglyceride accumulation. Excessive lipid accumulation in the glomerulus and renal tubules leads to podocyte dysfunction and damage to proximal tubular epithelial cells and tubular interstitial tissue (144). In addition, proteinuria in patients with diabetes may also serve as a carrier of fatty acids in urine. This leads to the accumulation of fatty acids in the kidney, thus exacerbating renal tubular injury in patients with diabetes (145). In OVX diabetic female rats, due to the lack of estrogen, lipid metabolism disorders occur, and fasting blood glucose levels and the insulin resistance value (HOMA-IR) were significantly increased compared with those in the control group (146).

Generally, glucose/lipid metabolic disorders may induce DN through oxidative stress, inflammation and hemodynamic changes. Therefore, the role of sex hormones in the modulation of these processes is the same as stated above.

**Figure 2:**
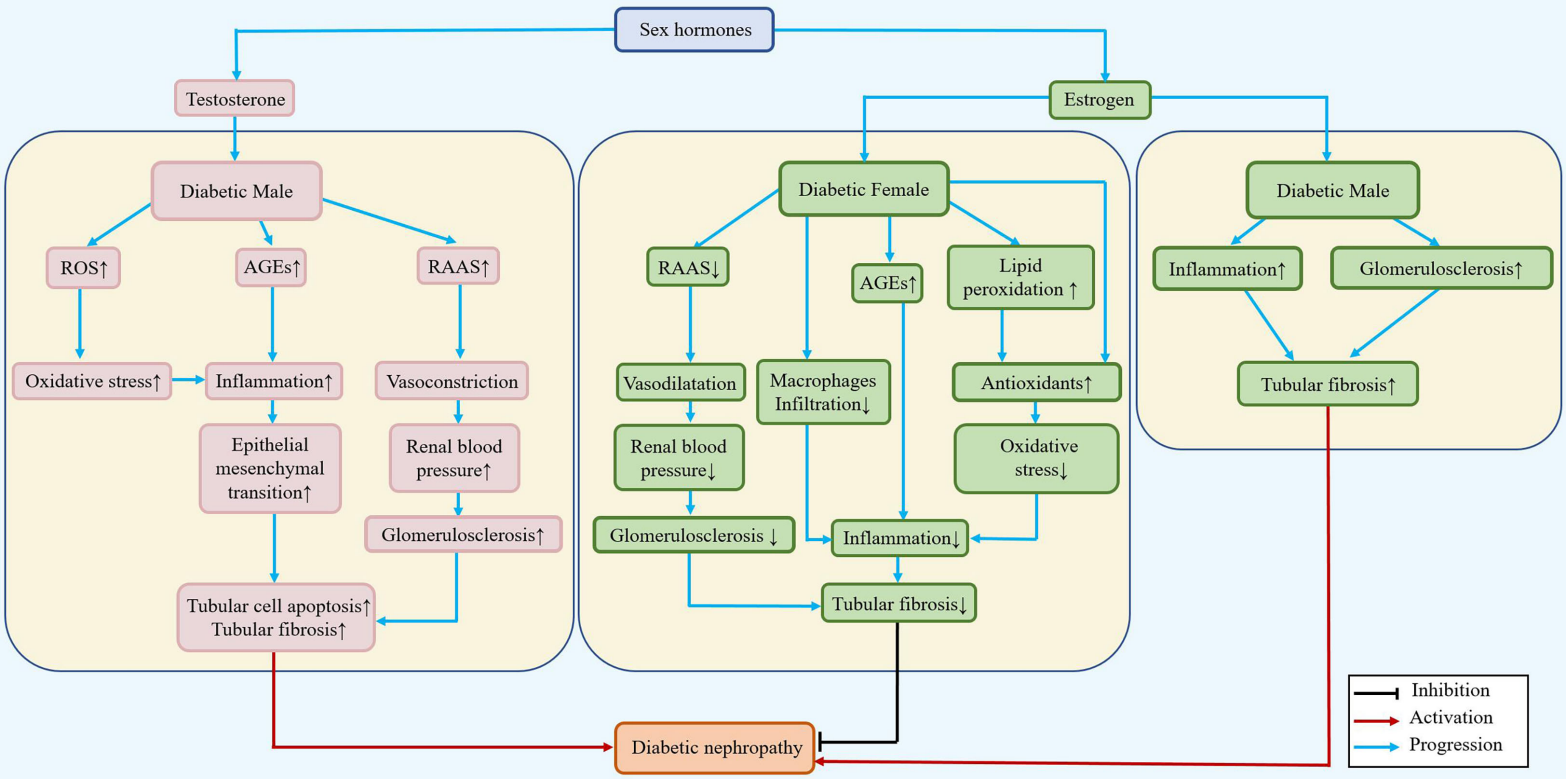
Differential roles of estrogen/testosterone in the pathogenesis of DN. During diabetes, testosterone can increase the renal blood pressure and inflammatory response, accelerate the epithelial mesenchymal transition, and lead to renal tubular fibrosis in males. In females, estradiol can reduce renal tubular fibrosis by reducing blood pressure, downregulating the inflammatory response and epithelial mesenchymal transition, and playing a renoprotective role.

The effects of sex hormones that may function in the occurrence of DN are illustrated in **Figure 2**, and the molecules affected by sex hormones in the progression of DN are listed in **Table 1**.

**Table 1:** Sex hormones affect the pathogenesis of DN and related molecules.

Sex hormones	Changes in the molecules involved in DN pathogenesis			Outcomes	Reference
	Oxidative stress	Inflammation	Renal haemodynamics		
Testosterone	STAT3↓ ROS↑	C-jun↑ MCP-1↑ TNF-α↑ CTGF↑ Type IV collagen↑ TGF-β↑	ATR1↑ 3β-HSD4↓ AngII↑	Fibrosis↑ Kidney injury↑	(60, 73, 93, 112–116, 131, 132)
Estrogen	Antioxidants (e. g. GSH-Px, GSH and SOD) ↑ Nox↓ ROS↓	Smad 2/3↓ Smad 6/7↑ CK2↓ Type IV collagen↓ TGF-β↓	eNOS↑ NO↑ ATR2↑ AngII↓	Fibrosis↓ Kidney injury↓	(73, 80, 94–96, 119–121, 130, 133–135, 138–142)

Annotation: STAT3, signal transducer and activator of transcription-3; ROS, reactive oxygen species; Nox, NADPH oxidases; GSH-Px, glutathione peroxidase; GSH, glutathione; SOD, superoxide dismutase; MCP-1, monocyte chemoattractant proteins-1; TNF-α, tumor necrosis factor-α; CTGF, connective tissue growth factor; TGF-β, transforming growth factor-β; CK2, casein kinase II; ATR1, angiotensin II receptors-1; AngII, angiotensin II; eNOS, endothelial nitric oxide synthase; NO, nitric oxide; ATR2, angiotensin II receptors-2. The symbol "↑" means upregulation.The symbol "↓" means downregulation.

## Effects of sex hormone replacement therapies for DN

### Effects of sex hormone replacement therapies in females with DN

Using E2 supplementation therapy for DN obtains good results in reducing kidney injury in women; for example, Szekacs et al. reported that in postmenopausal women with DN, estradiol supplementation reduces albuminuria (147). Raloxifene is a type of selective estrogen receptor modulator. It may attenuate glomerulosclerosis and albuminuria in women with DN and slow the progression of nephropathy (148–151). In addition, Bahaa et al. also found that progesterone treatment can attenuate DN in females (152). However, the risk or side effects of sex hormone therapies are nonnegligible. Eliassen et al. reported that E2 supplementation in premenopausal women increases their risk of breast cancer, but Dixon et al. found that raloxifene does not have side effects similar to those of E2 (149, 153). Moreover, the side effects of progesterone in the treatment of DN have been less frequently reported (152).

### Effects of sex hormone replacement therapies in males with DN

Using sex hormone therapy for males with DN has been less commonly reported. Qin Xu et al. found that DHT has a dose-dependent effect in DN male rats. DHT at low concentrations (0.75 mg) can partly ease the progression of nephropathy, while DHT at high concentrations (2.0 mg) has the opposite effects in the kidney (154).

Icariin is a recently discovered GPER agonist. Qi et al. reported that icariin has antioxidative stress and antifibrotic effects in DN male rats, but whether it has side effects is unclear and not reported (155).

**Table 2** summarizes the existing preclinical/clinical/animal experiments using sex hormone replacement therapies and their roles in the treatment of DN models.

**Table 2:** Sex hormone replacement therapies in DN.

Drug	Research category	Object	Method	Outcome	Reference
Estradiol	Clinical research	Postmenopausal women with DN	Oral estradiol (2mg/day) combined with norgestrel (0.5mg/day)	Albuminuria↓ CrCl↑	(147)
	Preclinical research/animal experiment	Female rats with DN	Estradiol pellets implanting after OVX (10μg/day)	Albuminuria↓ GSI↓ TIFI↓ Blood glucose level↓	(38)
	Preclinical research/animal experiment	db/db female mouse	Subcutaneous implantation of estradiol pellets after OVX(8.3μg/day)	UAE↓ Mesangial expansion↓ Fibronectin↓ Blood glucose level↓	(156)
Raloxifene	Clinical research	Postmenopausal women with DN	Oral (60mg/day)	Albuminuria↓ Risk of vertebral fracture↓ No effect on fasting blood glucose with short-term raloxifene treatment	(148, 150, 151)
	Preclinical research/animal experiment	Female rats with DN	Administering in the phytoestrogen-free chow (10mg/kg/day)	UAE↓ GSI↓ TITF↓ Type I/IV collagen↓ TGF-β↓ IL-6↓	(149)
	Preclinical research/animal experiment	db/db female mouse	Subcutaneous treatment (10mg/kg/day)	Mesangial area↓ TGF-β↓ Fibronectin↓	(156)
Progesterone	Preclinical research/animal experiment	Female rats with DN	Progesterone treatment after OVX (10mg/kg)	UACR↓ GSI↓ Fibronectin↓ ATR1↓ TGF-β↓	(152)
Dihydrotestosterone	Preclinical research/animal experiment	Male rats with DN	Dihydrotestosterone in low concentrations subcutaneous implantation (0.75mg/day)	UAE↓ Glomerular sclerosis↓ TITF↓ Type IV collagen↓ TGF-β↓ IL-6↓	(154)
	Preclinical research/animal experiment	Male rats with DN	Dihydrotestosterone in high concentrations subcutaneous implantation (2.0mg/day)	Opposite results compared with dihydrotestosterone in 0.75mg/day concentrations (low concentration)	(154)
Icariin	Preclinical research/animal experiment	Male rats with DN	Oral (80mg/kg)	MDA↓ Type IV collagen↓ TGF-β↓	(155)
	Preclinical research/animal experiment	Male rats with DN	Gavage (20, 40, and 80 mg/kg/day)	Blood urea nitrogen↓ Urine protein↓ Urinary creatinine↓ CrCl↑ TITF↓	(157)

Annotation: CrCl, creatinine clearance rate; GSI, glomerulosclerotic index; TIFI, the index of tubulointerstitial fibrosis; UAE, urinary albumin excretion; TITF, tubulointerstitial fibrosis; TGF-β, transforming growth factor-β; IL-6: interleukin-6; UACR, urinary albumin to creatinine ratio; ATR1, angiotensin II receptor 1; MDA, malondialdehyde. The symbol "↑" means upregulation.The symbol "↓" means downregulation.

## Conclusions

In summary, many studies have shown that the occurrence and progression of DN are closely related to sex hormones. Testosterone can exacerbate DN by activating the RAAS or phosphorylating C-jun to induce tubular fibrosis, so DN usually progresses faster in male patients than in female patients. Estradiol can upregulate the expression of eNOS and increase the level of NO to alleviate the vasoconstriction effect of AngII to reduce tubular fibrosis. In addition, estradiol can alter the level of Smad family members and reduce macrophage infiltration and CK2 activation to alleviate tubular fibrosis. Thus, estradiol is thought to play a protective role in DN. Along with that for new targets for treatment, understanding the effect of sex hormones will provide a new combined therapeutic strategy for DN. Particular challenges are presented and placed within the context of future treatments against DN.

## Author contributions

WW and DY conceived the manuscript. JL and DY drafted the manuscript. JL drew the figures. JL, WS and WW proofread the manuscript and made revisions. LL and XA collected the references. All authors contributed to the article and approved the submitted version.
